# Mal/SRF Is Dispensable for Cell Proliferation in *Drosophila*

**DOI:** 10.1371/journal.pone.0010077

**Published:** 2010-04-09

**Authors:** Barry J. Thompson

**Affiliations:** Cancer Research UK, London Research Institute, London, United Kingdom; University of Texas MD Anderson Cancer Center, United States of America

## Abstract

The Mal/SRF transcription factor is regulated by the level of G-actin in cells and has important roles in cell migration and other actin-dependent processes in *Drosophila*. A recent report suggests that Mal/SRF and an upstream regulator, Pico, are required for cell proliferation and tissue growth in *Drosophila*. I find otherwise. Mutation of Mal or SRF does not affect cell proliferation in the fly wing. Furthermore, I cannot reproduce the reported effects of Pico RNAi or Pico overexpression on body size. Nevertheless, I can confirm that overexpression of Pico or Mal causes tissue overgrowth specifically in the fly wing - where SRF is most highly expressed. My results indicate that Mal/SRF can promote tissue growth when abnormally active, but is not normally required for tissue growth during development.

## Introduction

The control of tissue growth in *Drosophila* requires the action of multiple signalling pathways that often have conserved roles in mammalian development and cancer [1], [2], [3]. It was recently reported that tissue growth in *Drosophila* also depends on a signalling pathway involving the lammelipodin homologue Pico and the Mal/SRF transcription factor [4]. This pathway has been well studied in mammalian cells, where it has been shown that high levels of G-actin in cells activate Mal and promote its translocation to the nucleus where it binds to and activates the SRF transcription factor [5]. Mal/SRF has important functions in cell migration and other actin-dependent processes in both *Drosophila* and mammals [6], [7], as well as regulating cell fate in the *Drosophila* wing [8], but only a single report by Lyulcheva *et al* claims that this transcription factor regulates tissue growth in *Drosophila* [4].

The evidence that Lyulcheva *et al* present that Pico, Mal and SRF regulate tissue growth in *Drosophila* is largely based on overexpression of Pico and Mal - which were reported to increase wing size [4]. In addition, overexpression of Pico was also reported to increase the size of the whole body, suggesting that this pathway might control growth in all tissues [4]. Finally, RNAi knockdown of Pico was found to reduce wing size and body size [4]. Lyulcheva *et al* concluded that Pico is required for tissue and organismal growth and that Pico acts via control of mitogenic SRF signalling [4].

Since no loss of function analysis of the requirement for Mal or SRF in cell proliferation or tissue growth has been performed, I decided to carry this out. Surprisingly, my results conflict with those reported by Lyulcheva *et al* and suggest that signalling through Mal/SRF is not required for cell proliferation or tissue growth during fly development. I also find that ectopic activation of Mal can stimulate tissue growth, but only in the fly wing, where SRF is most highly expressed.

## Results

**Figure 1:**
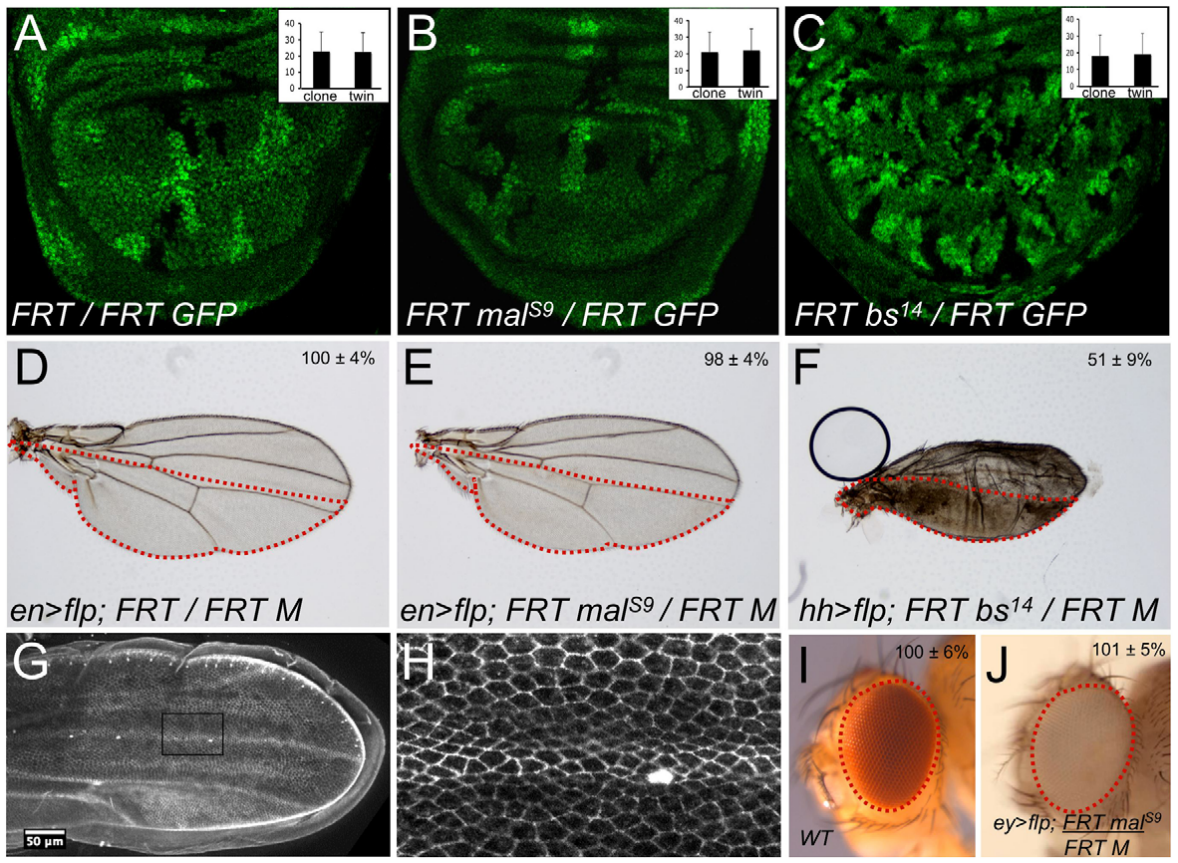
Mal/SRF is not required for cell proliferation in the fly wing or eye. (A) Clones of control cells (absence of GFP) and their twin spots (bright GFP) are roughly the same size, indicating normal rates of proliferation. (B) Clones of *mal^S9^* mutant cells (absence of GFP) and their twin spots (bright GFP) are roughly the same size, indicating normal rates of proliferation. (C) Clones of *bs^14^* mutant cells (absence of GFP) and their twin spots (bright GFP) are roughly the same size, indicating normal rates of proliferation. (D) A control wing containing wild-type cells in the posterior compartment. Genotype is indicated. (E) A wing containing *mal^S9^* mutant cells in the posterior compartment. Genotype is indicated. (F) A wing containing *bs^14^* mutant cells in the posterior compartment. Genotype is indicated. (G) A pupal wing expressing E-cad GFP. (H) A close up of a wing vein from (G). (I) A wild-type fly eye. (J) A *malS9* mutant fly eye, genotype indicated, is normally sized.

To test the requirement for the *mal* and *blistered* (*bs*, encoding SRF) genes in cell proliferation, I generated clones of cells marked by the absence of GFP in the developing fly wing with the *hs.flp/FRT* method. I find that wild-type clones, *mal^S9^* null mutant clones, and *bs^14^* null mutant clones all proliferate normally, reaching similar sizes as their twin-spot (bright GFP) clones (Fig. 1A–C). I then used *en.flp/FRT Minute* or *hh.flp/FRT Minute* methods to generate adult wings containing large clones that fill the entire posterior compartment. Wings with wild-type or *mal^S9^* mutant posterior compartments were normally sized (Fig. 1D,E). Wings with a *bs^14^* mutant posterior compartment exhibited a transformation of all cell types to vein fate - due to a well established requirement for SRF in vein patterning that is independent of Mal (Fig. 1F) [8]. Since vein cells are smaller than inter-vein cells, due to apical constriction, the size of the wing is reduced due to this morphological change (Fig. 1G,H). Wild-type eyes and *malS9* mutant eyes generated with the *ey.flp/FRT Minute* method were normally sized (Fig. 1I,J). These results show that Mal/SRF activity is not required for cell proliferation in the fly wing or eye.

**Figure 2:**
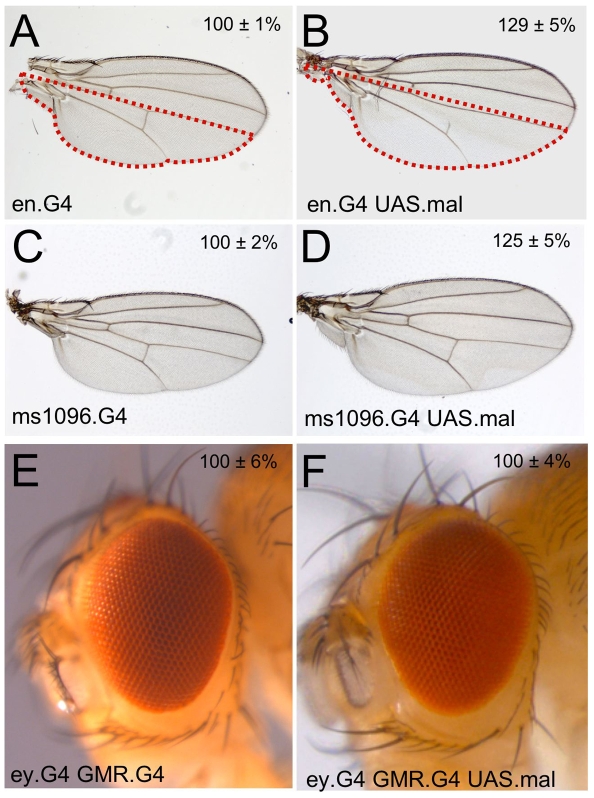
Overexpression of Mal causes overgrowth in the wing, but not the eye. (A) A control *en.Gal4* wing. (B) Overexpression of Mal in the posterior compartment with *en.Gal4* causes overgrowth. (C) A control *ms1096.Gal4* wing. (D) Overexpression of Mal in the whole wing with *ms1096.Gal4* causes overgrowth. (E) A control *ey.Gal4 GMR.Gal4* eye. (F) Overexpression of Mal in the eye with *ey.Gal4 GMR.Gal4* does not affect eye size.

I next overexpressed Mal in both the wing and the eye to examine its effect on tissue growth. I find that, as reported by Lyulcheva *et al*, overexpressed Mal causes overgrowth of the fly wing when driven in either the posterior compartment (Fig. 2A,B) or in the whole wing (Fig. 2C,D). In contrast, overexpression of Mal in the fly eye thoughout development does not cause tissue overgrowth (Fig. 2E,F). These results show that ectopically expressed Mal can induce tissue growth, but specifically in the fly wing.

**Figure 3:**
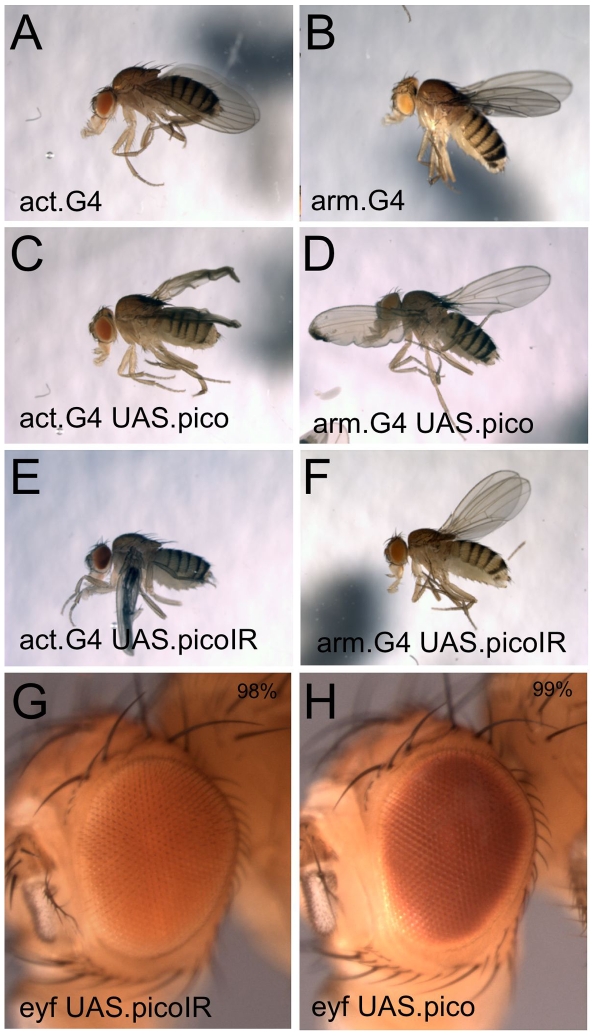
Pico is not required to regulate body size or eye size. (A) A control *act.Gal4* fly. (B) A control *arm.Gal4* fly. (C) Ubiquitous overexpression of Pico with *act.Gal4* does not change body size, but does affect wing morphology. (D) Ubiquitous overexpression of Pico with *arm.Gal4* does not change body size, but does affect wing morphology. (E) Ubiquitous RNAi knockdown of Pico with *act.Gal4* does not change body size, but does affect wing morphology. (F) Ubiquitous RNAi knockdown of Pico with *arm.Gal4* does not change body size, but does affect wing morphology. (G) RNAi knockdown of Pico in the eye with *ey.flp act>STOP>Gal4* produces normally sized eyes. (H) Overexpression of Pico in the eye with *ey.flp act>STOP>Gal4* produces normally sized eyes.

The above results caused us to question the Lyulcheva *et al* model that Pico regulates tissue and organismal growth via regulation of Mal/SRF. I therefore repeated the published experiments expressing *UAS.pico* or *UAS.pico-IR* (an RNAi-inducing inverted repeat) transgenes obtained from the authors in the whole fly. I find that neither transgene affects body size or average weight (act.G4 control  =  0.0025g; act.G4 UAS.pico  =  0.0024 g; act.G4 UAS.pico-IR  =  0.0027 g) compared with control animals (Fig. 3A–F). Expression of the same transgenes thoughout eye development also had no effect on eye size (Fig. 3G,H). These results suggest that Pico is not a general regulator of tissue or organismal growth in *Drosophila*.

**Figure 4:**
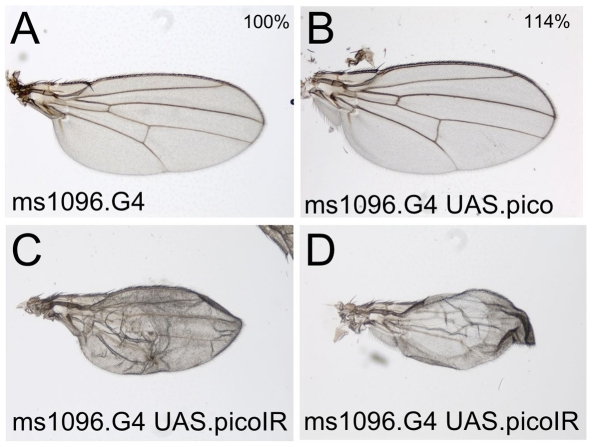
Pico is required for morphogenesis in the wing and can drive overgrowth when overexpressed. (**A**) A control *ms1096.Gal4* wing. (**B**) Overexpression of Pico with *ms1096.Gal4* drives overgrowth, similar to overexpression of Mal. (**C,D**) RNAi knockdown of Pico with *ms1096.Gal4* causes blisters (C) or crumpling (D) but does not strongly reduce wing size.

My experiments with Pico overexpression and RNAi knockdown in the whole body revealed some effects on wing development. I therefore specifically expressed *UAS.pico* or *UAS.pico-IR* in the fly wing with a strong wing Gal4 driver. I find that overexpression of Pico causes tissue overgrowth (Fig. 4A,B), similar to overexpression of Mal (Fig. 2C,D), confirming previous work. However, RNAi knockdown of Pico does not cause a clean tissue undergrowth phenotype but instead causes crumpling of the wing, suggesting that a defect in morphogenesis, rather than growth, may be responsible for reduced wing size. These results suggest that Pico is required for wing morphogenesis and that, like Mal, overexpression of Pico can induce tissue overgrowth specifically in the wing.

## Discussion

My results conflict with a previous report by Lyulcheva *et al* suggesting that Pico is an essential regulator of tissue and organismal growth that acts by regulating Mal/SRF [4]. I instead find that Mal and SRF are dispensable for cell proliferation and tissue growth in *Drosophila*. SRF does affect the final size of the wing, but via regulation of cell fate rather than cell proliferation. Any requirement for Pico in cell proliferation is therefore not mediated by Mal/SRF but is rather an indirect effect of Pico, most likely via its role in maintaining actin-dependent cellular morphology. Consistent with this, analysis of *pico* mutant clones indicates that the mutant cells are extruded from the epithelium [4].

Although Mal/SRF is dispensable for tissue growth, my results confirm that ectopic activation of this transcription factor is capable of causing tissue overgrowth in the wing. Overexpression of Pico or Mal is sufficient to induce Mal/SRF driven overgrowth of the wing, but has little effect elsewhere in the adult body. This is consistent with previous reports that SRF is not uniformly expressed in all fly tissues but appears to be expressed in a specific pattern in the developing wing [8]. Thus, in imaginal epithelia where SRF is expressed, ectopic activation of Mal can drive tissue overgrowth.

The SRF expression pattern corresponds to the future intervein wing cells, with SRF absent in developing veins [8]. Loss of SRF (in *bs* mutants) causes intervein cells to transform into vein cells [8]. Loss of Mal does not affect vein patterning, consistent with the view that SRF promotes intervein fate independently of Mal.

In conclusion, the normal function of SRF is to pattern the fly wing, however, in the presence of ectopically active Mal, SRF can also promote tissue overgrowth. Mal/SRF might therefore have a role in promoting tumour growth in humans and it will be interesting to determine whether Mal is ectopically active tumours and whether it is sufficient to drive tissue growth in mouse models.

## Materials and Methods

### *Drosophila* genetics

*FRT malS9* and *FRT bs14* were obtained from P. Rorth [6]. *en.Gal4 UAS.Flp* and *hh.Gal4 UAS.Flp* were obtained from J-P. Vincent [9]. *UAS.mal*, *UAS.pico*, and *UAS.picoIR* stocks were obtained from D. Bennett [4]. Other stocks were obtained from the Bloomington *Drosophila* stock centre.

Fly crosses were performed at 25°C and adult flies were dissected and examined by bright field microscopy. Wings were fixed in ethanol, dipped in distilled water and then mounted on glass slides in Hoyer's medium. Imaginal discs and pupal wings were examined with a Leica SP5 laser-scanning confocal microscope.

### Quantification

Clone sizes were quantified based on the number of cells/nuclei per clone (observed by DAPI staining) and average cell number per clone and standard deviations were graphed using Microsoft Excel for both clones and twin spots.

Wing sizes were quantified with ImageJ (the pixel measurement function) for multiple wings from both control and test animals. Average wing size and standard deviations were calculated with Microsoft Excel and expressed as a percentage of wild-type size ( = 100%).

Body weight was measured by collecting a defined number of female flies in an eppendorf tube and measuring their weight with a fine balance relative to an empty eppendorf tube. Average fly weight was determined by dividing the total weight by the number of flies.
